# Association between genotypic diversity and biofilm production in group B *Streptococcus*

**DOI:** 10.1186/s12866-016-0704-9

**Published:** 2016-05-20

**Authors:** Robert E. Parker, Clare Laut, Jennifer A. Gaddy, Ruth N. Zadoks, H. Dele Davies, Shannon D. Manning

**Affiliations:** Department of Microbiology and Molecular Genetics, Michigan State University, East Lansing, MI USA; Tennessee Valley Healthcare System, Department of Veterans Affairs, Nashville, TN USA; Department of Medicine, Vanderbilt University, Nashville, TN USA; Institute for Biodiversity, Animal Health and Comparative Medicine, University of Glasgow, Glasgow, UK; Moredun Research Institute, Penicuik, UK; University of Nebraska Medical School, Omaha, NE USA

## Abstract

**Background:**

Group B *Streptococcus* (GBS) is a leading cause of sepsis and meningitis and an important factor in premature and stillbirths. Biofilm production has been suggested to be important for GBS pathogenesis alongside many other elements, including phylogenetic lineage and virulence factors, such as pili and capsule type. A complete understanding of the confluence of these components, however, is lacking. To identify associations between biofilm phenotype, pilus profile and lineage, 293 strains from asymptomatic carriers, invasive disease cases, and bovine mastitis cases, were assessed for biofilm production using an in vitro assay.

**Results:**

Multilocus sequence type (ST) profile, pilus island profile, and isolate source were associated with biofilm production. Strains from invasive disease cases and/or belonging to the ST-17 and ST-19 lineages were significantly more likely to form weak biofilms, whereas strains producing strong biofilms were recovered more frequently from individuals with asymptomatic colonization.

**Conclusions:**

These data suggest that biofilm production is a lineage-specific trait in GBS and may promote colonization of strains representing lineages other than STs 17 and 19. The findings herein also demonstrate that biofilms must be considered in the treatment of pregnant women, particularly for women with heavy GBS colonization.

**Electronic supplementary material:**

The online version of this article (doi:10.1186/s12866-016-0704-9) contains supplementary material, which is available to authorized users.

## Background

Group B *Streptococcus* (GBS), or *Streptococcus agalactiae*, is a leading cause of neonatal sepsis and meningitis worldwide [1, 2]. Originally identified as the etiological agent in bovine mastitis, GBS is present as a commensal in the gastrointestinal and urogenital tracts in 15–30 % of healthy adults [3]. Human cases of invasive GBS disease were reported in the early 1900’s where GBS was identified as the primary cause of neonatal infections, with up to a 50 % mortality rate and devastating long term effects for survivors [4, 5]. GBS is also responsible for soft tissue infections in the elderly and immunocompromised individuals [6]. In most neonatal infections, the pathogen is vertically transmitted before or during birth, however, in adults there are implications of transmission due to exposure to either infected humans or other animals [7–9]. Understanding the interplay of factors driving transmission of and persistent infection with GBS is essential to the development of treatments leading to the prevention of disease.

Colonization and persistence are critical for GBS-mediated disease in humans. Guidelines established in 1996 in the United States recommended screening for maternal colonization during the late third trimester followed by intrapartum antibiotic prophylaxis of GBS-positive women during labor [4]. The institution of these guidelines has resulted in a dramatic decrease in the incidence of neonatal early onset disease (EOD), or cases within the first week of life. Studies have shown, however, that re-colonization of the mother occurs in up to 65 % of cases following antibiotic treatment, which may explain why having a previous baby with invasive GBS disease is a risk factor for neonatal disease, and no changes have been reported in the incidence of late onset disease (LOD), or infections occurring up to 3 months of age [10–12]. Application of multilocus sequence typing (MLST) targeting seven reference genes has identified specific sequence types (STs) and clonal complexes (CCs) to be associated with maternal colonization as well as neonatal disease. CCs 1 and 23, for instance, have previously been linked to asymptomatic colonization, while CCs-17 and -19 were found to predominate among neonates. Nonetheless, differences in CC distributions have been noted across populations [13–16] as have risk factors. In addition to complications during labor, heavy GBS colonization of the mother was suggested to be a risk factor for neonatal infections and preterm birth [17], though few studies have explored whether the density of maternal colonization is linked to specific bacterial factors.

Several factors have been found to be important for GBS colonization, the first step in pathogenesis. Proteins shown to facilitate binding to host cell surface components include the laminin-binding protein (Lmb), fibrinogen binding proteins (FbsA, FbsB, and ScpB), serine-repeat rich proteins (Srr-1 and Srr-2), and pili [18–24]. Similarly, the chemical composition and antigenic variation of the polysaccharide capsule has been linked to virulence while survivability in different environments and biofilm formation were also suggested to be important, particularly in the case of persistent colonization [25–29]. A biofilm is defined as an aggregation of cells in a distinctly sessile state surrounded by a self-produced matrix composed of polysaccharide as well as protein and DNA [30]. For some bacterial pathogens, biofilm production is an important virulence determinant that has been linked to colonization and disease progression [30, 31]. Biofilms offer protection in harsh environments that can include antimicrobials, extreme pH, and immune cells, thereby promoting the maintenance of a bacterial population that can contribute to chronic infection and heavy colonization [32–35]. The specific environmental conditions found within biofilms can also exert a selective pressure that can enhance pathogenicity via the rise of phenotypic and genotypic variants [35, 36]. Additional information on GBS biofilms was published in a recent review by Rosini and Margarit [37]. Previous studies have shown that pili play an important role in biofilm formation in GBS, and each GBS genome encodes one or two distinct pilus islands (PI), PI-1 and PI-2 [20, 38, 39]. PI-1 has been observed in high frequency among human strains combined with one of two genetically distinct PI-2 variants, PI-2a and PI-2b [40, 41], yet the effect of PI sequence diversity on biofilm production has not been addressed. Because the role of biofilms in GBS-mediated disease is not known, and this phenotype has been observed to vary between isolates, we sought to characterize biofilm production across strains to identify biofilm determinants including isolation source, phylogenetic lineage, and variability in both presence and sequence of pilus loci [25, 26]. Furthermore, we sought to determine whether allelic variation within genes encoding the PIs impacts biofilm formation, as well as attachment to host cells, particularly among genotypes associated with neonatal disease. Because GBS pili have been posited as potential vaccination targets due to their importance in biofilms and adherence to and invasion of host cells, further understanding of allelic diversity and pilus-associated phenotypes could guide the development of new prevention strategies [40].

## Results

### Source and genotype are predictive of biofilm phenotypes

**Fig. 1:**
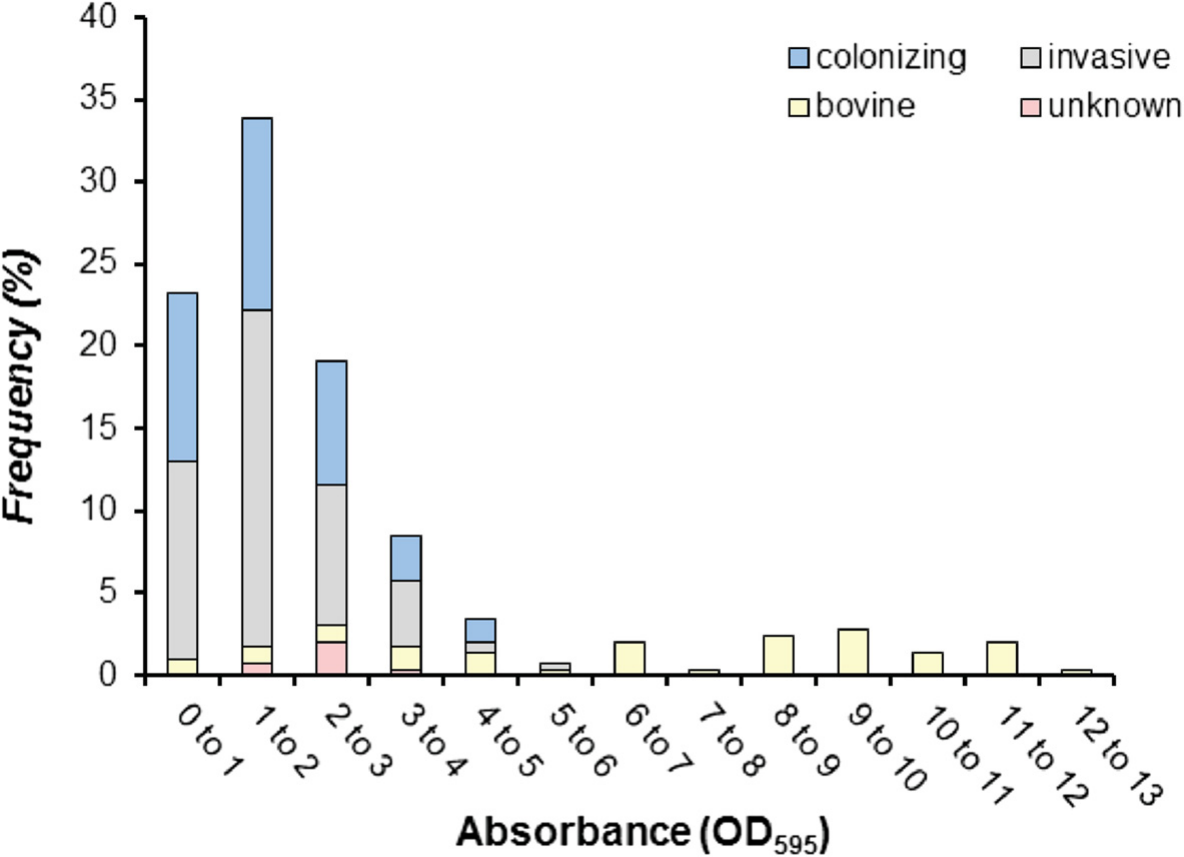
Source, frequency, and strength of biofilm production among 293 group B streptococcal strains by biofilm absorbance (OD_595_). Bar height represents the percentage of isolates within each absorbance category listed on the *x-axis*. Strong biofilm producers were classified as having an OD_595_ of 1.8 or greater

Assaying biofilm production in 293 strains, including 242 human and 51 bovine strains resulted in a range of absorbance values from 0.1 to 12.3 (Fig. 1). Using the median absorbance value of 1.8 to classify the biofilm phenotype, 148 (50.5 %) strains were categorized as strong biofilm producers and 145 (49.5 %) were designated weak biofilm producers. In all, there was considerable variation by source with human-derived strains having decreased levels of biofilm production relative to the bovine-derived strains. A total of 138 (57.0 %) of the 242 human-derived strains formed a weak biofilm compared to only six (11.8 %) of the 51 bovine-derived strains. Indeed, the bovine strains were significantly more likely to form strong biofilms relative to the human strains overall (Odds ratio (OR): 10.0; 95 % confidence interval (CI): 4.09, 24.21; *p* < 0.0001). The average absorbance (OD_595_) for human strains was 1.7 (range: 0.1 to 5.0), while the average OD_595_ for bovine strains was 6.3 (range: 0.6 to 12.3). Among human-derived strains, an association was also observed by source, as maternal colonizing strains were more likely to be strong biofilm producers compared to neonatal invasive strains (OR: 1.8; 95 % CI: 1.02, 3.06; *p* = 0.04). A total of 45 of the 98 (45.9 %) maternal colonizing strains had a strong biofilm phenotype relative to only 32.5 % (*n* = 39) of the 120 invasive strains from newborns with sepsis or meningitis.

**Fig. 2:**
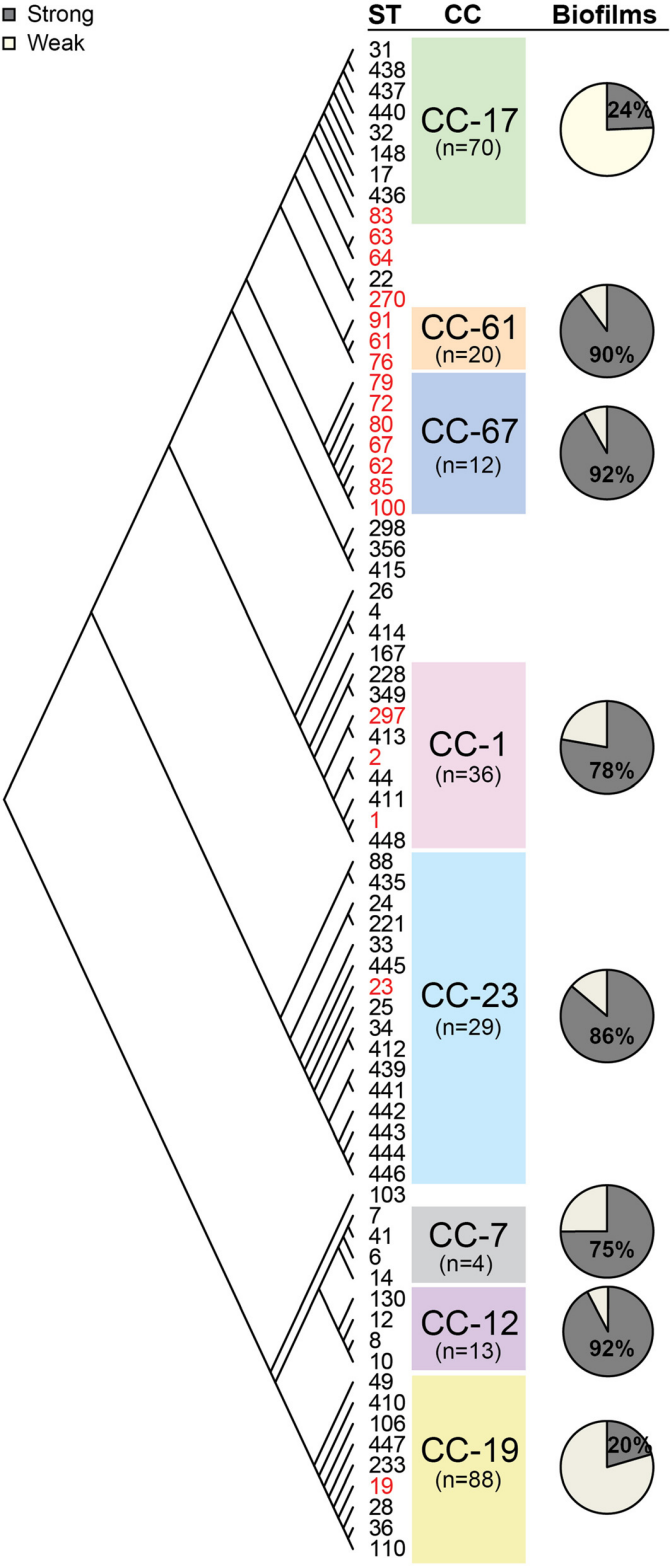
Variation in biofilm production among group B *Streptococcus* strains representing distinct phylogenetic lineages. The Neighbor-joining phylogeny grouped the 73 sequence types (STs) into eight clonal complexes (CCs), which are represented with different colors. Bovine strains are indicated in *red* and the frequency (%) of strains forming a strong (*dark grey*) versus weak (*light grey*) biofilm is shown within each CC as individual pie charts

To examine phenotypic variation by genotypes, biofilm production levels were compared between STs and CCs (Fig. 2). Interestingly, the majority (87.3 %) of the weak biofilm producers belonged to CC-17 and CC-19; 75.7 % (*n* = 53) and 79.6 % (*n* = 70) of the strains belonging to these two lineages, respectively, were classified as weak. Although both lineages were overrepresented in the analysis, it is notable that no other lineages had weak biofilm producers outnumbering the strong producers. Among all 158 CC-17 and CC-19 strains combined, 77.8 % (*n* = 123) formed weak biofilms compared to only 15.6 % of the 135 strains belonging to all other CCs (*X*^*2*^ = 113.0, *p* < 0.0001). By contrast, the lineages that were exclusively comprised of bovine strains (e.g., CCs 61 and 67) were most frequently classified as strong biofilm producers. Only three strains among all 32 strains representing CC-61 and CC-67 had weak levels of biofilm production. It is important to note that some CCs such as CC-23 and CC-1, which mostly contained human-derived strains, also contained a subset of three and four bovine-derived strains, respectively. After excluding these bovine strains from the analysis, however, both CCs 23 and 1 were still overrepresented with strong biofilm producing strains. Similarly, although there was only one representative of each, the bovine-derived strains representing CC-17 and CC-19 formed strong biofilms unlike the remainder of the strains within these two lineages. When the 51 bovine isolates were compared to 75 human isolates comprising the lineages previously associated with asymptomatic carriage (e.g., CCs 1, 7, 12, and 23), there was no significant difference in biofilm phenotype frequencies.

In addition, we examined associations between disease presentation and biofilm phenotype for 70 (62.5 %) and 42 (37.5 %) isolates recovered from neonates with EOD and LOD, respectively. These isolates represented CCs 1 (*n* = 5), 12 (*n* = 2), 17 (*n* = 53), 19 (*n* = 40), and 23 (*n* = 8) as well as singletons (*n* = 4). When all 112 isolates were examined together, there was no association between biofilm phenotype and disease onset; however, when stratified by CC, weak biofilm-formers belonging to CCs 17 and 19 were significantly more likely to cause EOD. Among the EOD cases, strong biofilm producers belonging to lineages other than CCs 17 and 19 were 28.7 times more likely to cause EOD (CI: 6.75, 121.69; *p* < 0.0001) than strains of CCs 17 and 19. A similar comparison could not be examined for LOD cases as all were caused by CC-17 strains in this study.

### Biofilm production in GBS is influenced by PI occupancy and variation in PI genes

**Fig. 3:**
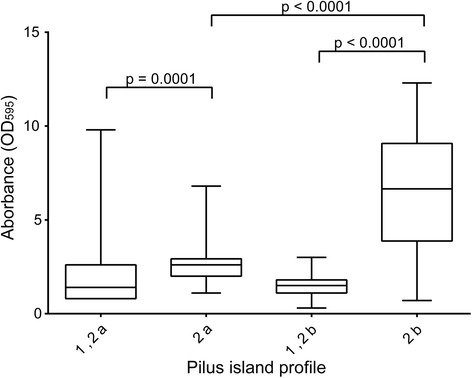
Biofilm absorbance among group B streptococcal strains with different pilus island (PI) profiles. *Center line* of boxes is average absorbance, *boxes* represent the middle two quartiles, and *whisker bars* represent highest and lowest quartiles

Biofilm production varied across strains with different PI profiles. Strains containing a PI-2 variant alone were significantly more likely to produce strong biofilms compared to strains with a PI-2 variant as well as PI-1 (OR: 10.4; 95 % CI: 4.91, 22.00; *p* < 0.0001). Weak biofilm production was more common in strains with both PI-1 and either PI-2 variant as 54.9 % of the 142 PI-1/PI-2a-positive strains and 70.3 % of the 81 PI-1/2b-positive strains formed weak biofilms. Significantly more PI-1/2b strains, however, formed weak biofilms compared to the PI-1/2a strains (OR: 1.9; 95 % CI: 1.09, 3.48; *p* < 0.02). Although an equal percentage of strains with exclusively PI-2a (*n* = 22; 84.6 %) or PI-2b (*n* = 39; 88.6 %) were capable of forming strong biofilms (*p* = 0.63), differences were noted in the absorbance values (Fig. 3). Specifically, strains with PI-2a alone had a mean absorbance value of 2.6 ± 1.0 compared to strains with PI-2b alone (6.5 ± 3.2; Mann-Wilcox test, *p* ≤ 0.0001). Strains with PI-2a alone also had a significantly higher mean absorbance value than strains with both PI-1 and PI-2a (1.9 ± 1.5; Mann-Wilcox test, *p* ≤ 0.0001), which also was true for strains containing both PI-1 and PI-2b (1.5 ± 0.6) relative to those with PI-2b alone (Mann-Wilcox test, *p* ≤ 0.0001). Because all strains with PI-2b alone were recovered from bovines, a comparison could not be made between PI-2b- and PI-2a-positive strains from humans.

To examine the association between genetic variation in PI genes and biofilm formation, we stratified biofilm strength by alleles detected in *gbs59*, the ancillary pilus protein in PI-2a, and *san1519*, the PI-2b adhesin. Variable biofilm production levels were observed among strains with different alleles of both genes. For *gbs59*, six alleles were identified and five of these six alleles (alleles 2-6) were significantly more common in strong biofilm producers (OR: 20.6; 95 % CI: 9.27, 45.62; *p* ≤ 0.0001). Only the *gbs59* allele 1, which predominated in 89 of the 168 PI-2a-positive strains, was more frequently detected in strains that formed weak biofilms (Table 1). The majority (*n* = 87; 97.8 %) of strains containing *gbs59* allele 1 belonged to CC-19, and more of these strains were recovered from neonates (*n* = 45; 52 %) than pregnant women (*n* = 39; 44.8 %); one strain originated from a bovine. Indeed, the neonatal CC-19 strains containing the *gbs59* allele 1 were significantly less likely to form strong biofilms relative to the maternal CC-19 strains with other alleles (*p* ≤ 0.02). Similar findings were observed for PI-2b, which were predominantly represented by CC-17. Strains with *san1519* allele 2 were significantly more likely to form weak biofilms relative to strains with *san1519* alleles 1 and 3 (OR: 17.3; 95 % CI: 6.99, 42.81; *p* ≤ 0.0001). No difference in frequency was observed for CC-17 strains with *san1519* allele 2 between neonates and pregnant women (Fisher’s exact *p* ≤ 1.0). Among the 32 bovine strains with *san1519* allele 3 from the bovine specific lineages, CCs 61 and 67, most (*n* = 29; 90.6 %) formed strong biofilms.

**Table 1:** Frequency of strong biofilm production among strains with distinct pilus island (PI) alleles and PI-1

Pilus island allele (a)	Total number of strains	Strong biofilm (%), n	Clonal complexes represented (n)	PI-1 presence (%)
PI-2a, a1	89	21.4 (*n* = 19)	19 (*n* = 87), 1 (*n* = 1), 12 (*n* = 1)	98.9
PI-2a, a2	16	87.5 (*n* = 14)	23 (*n* = 8), 1 (*n* = 7), S (*n* = 1)	93.8
PI-2a, a3	5	100.0 (*n* = 5)	12 (*n* = 3), 1 (*n* = 1), 7 (*n* = 1)	60.0
PI-2a, a4	6	83.3 (*n* = 5)	S (*n* = 5), 1 (*n* = 1)	66.7
PI-2a, a5	21	90.5 (*n* = 19)	23 (*n* = 17), S (*n* = 2), 19 (*n* = 1), 7 (*n* = 1)	14.3
PI-2a, a6	31	77.4 (*n* = 24)	1 (*n* = 17), 12 (*n* = 9), 23 (*n* = 4), S (*n* = 1)	93.6
PI-2b, a1	11	72.7 (*n* = 8)	1 (*n* = 8), 7 (*n* = 2), S (*n* = 1)	100.0
PI-2b, a2	69	21.7 (*n* = 16)	17 (*n* = 69)	100.0
PI-2b, a3	45	86.7 (*n* = 39)	61 (*n* = 20), 67 (*n* = 12), S (*n* = 11), 1 (*n* = 1), 17 (*n* = 1)	2.2

*S* singleton

### Multivariate analysis identifies source and genotype as important predictors of enhanced biofilm production

To further identify predictive features of strong biofilm production in GBS, we conducted a multivariate analysis including the following variables: pilus profile, source, and genotype. Among all GBS strains examined, only bovine source was moderately associated with strong biofilm production (OR: 3.6; 95 % CI: 0.92, 13.80; *p* = 0.07). A positive association was observed for the presence of PI-2a, but it was also not statistically significant (OR: 2.7; 95 % CI: 0.75, 9.90; *p* = 0.13) when adjusted for strain source and genotype. After excluding bovine strains from the analysis (Table 2), the presence of PI-2a remained associated with strong biofilm production; however, the association was still insignificant. Importantly, strains representing both CCs 17 and 19 were significantly less likely to form strong biofilms after controlling for source (invasive versus colonizing) and presence of PI-2a relative to other PI combinations. Unlike the univariate analysis, strains belonging to CCs 1 and 23 were not more likely to produce strong biofilms in the multivariate analysis.

**Table 2:** Multivariate analysis of characteristics associated with strong biofilm production in human-derived strains

Characteristics	Adjusted OR^a^ (95 % CI)	*p*-value*
Pilus Island (PI)		
Other PI combinations	1.0	–
PI-2a presence	4.0 (0.85,19.02)	0.08
Clonal complexes (CCs)		
Other CCs	1.0	–
CC-1	0.7 (0.18, 3.02)	0.66
CC-23	1.0 (0.19, 5.46)	0.99
CC-17	0.2 (0.03, 1.01)	0.05
CC-19	0.04 (0.01, 0.14)	<0.0001
Strain source		
Asymptomatic colonization	1.0	–
Invasive disease	1.2 (0.57, 2.33)	0.68

^a^ *OR* odds ratio, *95 % CI* 95 % confidence interval

*Walds Chi-square test

### The CC-17 lineage displays decreased association with Telomerase-immortalized Human Endothelial Cells (T-HESCs)

**Fig. 4:**
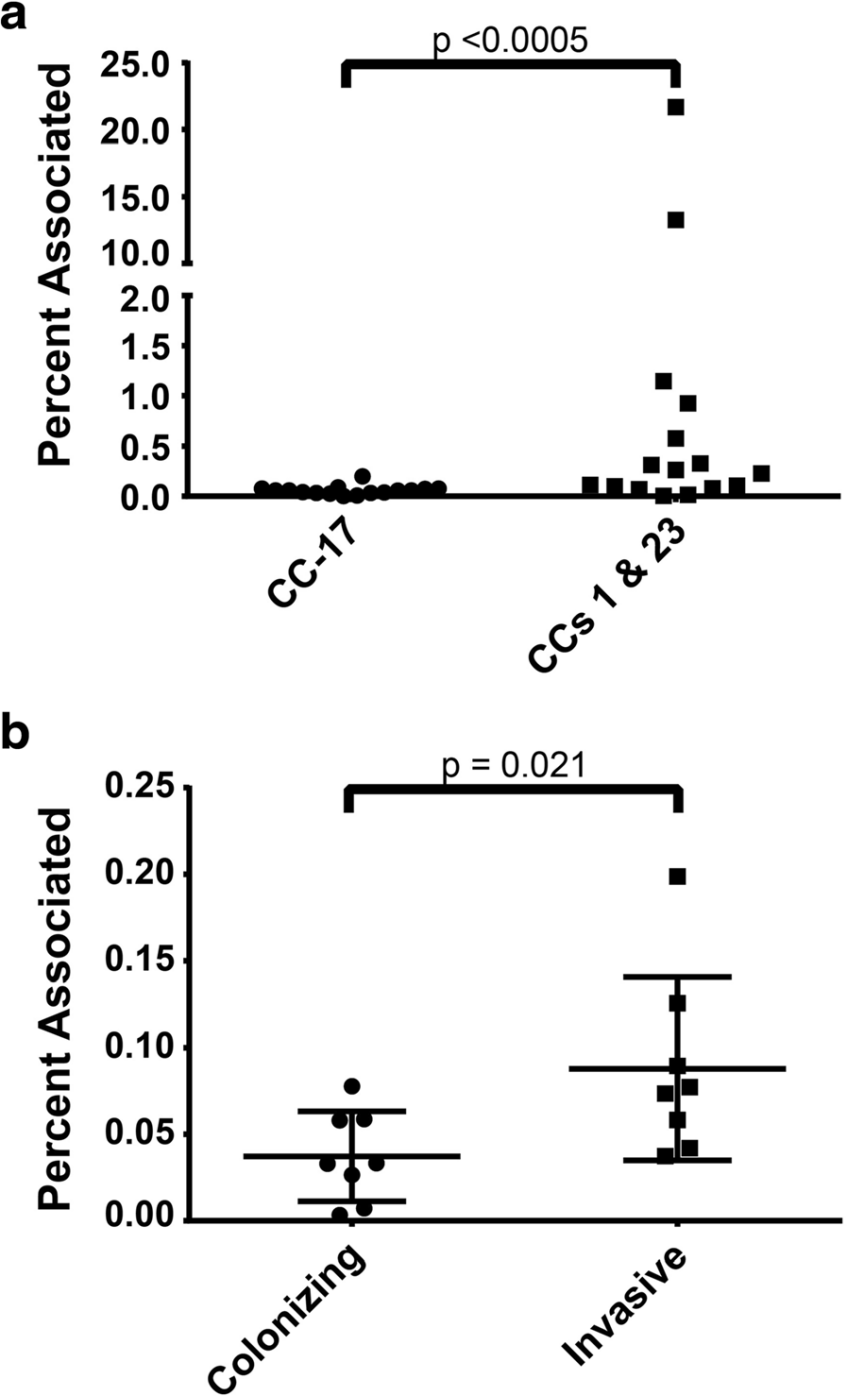
Variation in bacterial association with decidualized T-HESC. Percent association among **a**) 32 strains representing distinct phylogenetic lineages or clonal complexes (CCs); and **b**) 16 strains belonging solely to CC-17 by source. Individual data points represent the average of three experiments with the *largest horizontal lines* and error bars representing the mean of data points and standard deviations, respectively

Variation in the ability to associate with decidualized T-HESC was observed among the 32 strains chosen to represent CCs 1, 17, and 23 with different biofilm phenotypes. The 16 CC-17 strains evaluated had T-HESC attachment levels between 0.003 and 0.199 % with an average of 0.062 ± 0.048 %. The eight CC-1 strains had slightly greater association levels to T-HESC (0.015 to 1.145 %) than the CC-17 strains, whereas association levels for the eight CC-23 strains were highly variable (range: 0.004 to 21.68 %). Interestingly, association with T-HESCs by CC-17 strains was significantly decreased when compared to both CCs 1 and 23 combined with averages of 0.057 and 0.245 %, respectively (Mann-Wilcox test, *p*-value < 0.0005) (Fig. 4a). After stratifying association levels by biofilm production, no difference was observed within or between lineages. When source was considered, however, CC-17 strains from invasive disease cases associated with T-HESCs at higher levels than CC-17 maternal colonizing strains, with averages of 0.077 and 0.037 %, respectively. (Mann-Wilcox test, *p*-value <0.03) (Fig. 4b).

## Discussion

Because colonization of the host, or mother in the case of neonatal infection, is currently the primary predictor of GBS transmission and subsequent disease development [3, 42], we examined biofilm production in strains recovered from patients with invasive disease, individuals with asymptomatic colonization, and bovines with mastitis. Through this study we have demonstrated that biofilm production varies considerably across this diverse set of GBS strains. The phenotypic variation observed is in accordance with previous studies reporting OD_595_ values ranging from < 0.1 to >12 for GBS and other pathogens [25, 26, 43–45]. The OD_595_ value of 1.8 was used to distinguish between weak and strong biofilm production. This value is more conservative and higher than the OD_595_ values of 0.5 and 0.65 used in two prior studies with a similar biofilm assay, but it is lower than the value of 3.0 used in another study with a modified assay [25, 26, 46]. In our study, the distribution of OD_595_ values was not bimodal and hence, the 1.8 cut-off value was used to optimize sensitivity and specificity for the classification of weak versus strong biofilm producers. It is important to note, however, that any cut-off value carries a degree of subjectivity. In addition, differences between studies may be due to the media or type of plates used for the assays as both can alter the strength of a developing biofilm. In our study, we used THB plus 1 % glucose and tissue-culture treated polystyrene plates compared to TSB or untreated polystyrene plates in other studies [26, 46]. Because all strains were examined in similar conditions, our results are internally comparable, though external comparisons cannot be reliably made to data generated in other studies. It is important to note that we also assessed biofilm production in a subset of strains in other conditions including THB without glucose and in differing pHs as well as in the T-HESC infection media. The same trends were observed between isolates in these conditions, though the absorbance values were lower overall (data not shown).

Among all 293 strains examined, those strains originally recovered from bovines were capable of forming the strongest biofilms relative to the strains isolated from humans. Because there was no difference in biofilm phenotypes between strains representing the colonizing human lineages and the bovine lineages, these data suggest that strong biofilm production is more important for colonization in humans and bovines when compared to neonatal disease. It is therefore possible that unique genes or allelic variation within specific genes present in the bovine and human colonizing lineages, but lacking in invasive human strains, are important for biofilm phenotypes [47]. For example, we found that the human-derived strains possessing both PI-1 and either PI-2 variant had reduced biofilm production compared to those strains without PI-1. These data suggest an inhibitory effect of PI-1, an operon encoding a specific pilus type that is absent in bovine-derived strains belonging to the bovine-specific lineages [41]. Although a previous study found no effect on biofilm production in a PI-1 knockout [46], only one serotype Ia GBS strain was examined. Hence, it is possible that strains with varying genetic backgrounds, such as those belonging to CC-17 or CC-19 may behave differently when PI-1 is deleted. Similar to findings from a prior study [46], allelic variation within the genes comprising each PI may contribute to variation in the level of biofilm production observed. We found that specific alleles of *gbs59* and *san1519* were linked to strong biofilm formation while *san1519* allele 2, found exclusively in CC-17 strains, was associated with weak biofilm production. Since *san1519* allele 3 was restricted to the bovine strains [41], then this may also explain the association with strong biofilms. Despite these associations, future studies should focus on assessing the role of sequence variation on pili functionality and biofilms as other factors unique to specific lineages are also likely to be important. Given the proposed development of pilus-based vaccines for the treatment of GBS-mediated disease, these data highlight the importance of considering sequence variation in future vaccine development efforts similar to pilus-based vaccines targeting fimbriae in *Escherichia coli* and *Salmonella enterica*.

Another possible explanation for the increased biofilm production of the bovine-derived lineages is the presence of the lactose metabolism operon, Lac.2. This operon has been detected in the majority of bovine genomes, and upregulation of genes within the operon as well as genes important for glucose metabolism, have been documented following growth in milk [47, 48]. A prior study of *Streptococcus mutans* demonstrated enhanced biofilm production in the presence of lactose, a key component of milk [49]. Although it is possible that the ability to metabolize lactose and form strong biofilms is important for GBS survival in the bovine mammary gland to counteract the regular flow of milk, our in vitro biofilm assays were conducted without the addition of lactose. These data therefore suggest that other genes or gene combinations are more important for biofilm production in vitro. A prior study conducted by Ebrahimi et al. [50] also showed biofilm production to be a common feature of bovine strains in vitro, and hence, future studies should focus on mutagenesis of genes unique to bovine strains to determine their impact on biofilm formation and disease development in vivo.

In contrast to the consistently strong levels of biofilm production observed for the bovine strains, biofilm levels were highly variable between human-derived strains. Despite this variation, we found that strains of CC 17 and 19, the two CCs most commonly associated with invasive neonatal disease [14, 15], had significantly decreased levels of biofilm production compared to other CCs even after adjusting for PI profile and source. Likewise, we identified increased biofilm production in all but one of the bovine-derived strains representing lineages that were previously associated with asymptomatic carriage in humans [14]. Although the latter associations were less clear in the multivariate model, these findings indicate a correlation between weak biofilm production and increased pathogenicity. Furthermore, strains from neonates with invasive disease were more likely to form weak biofilms compared to colonizing strains recovered from pregnant women, which is a similar trend as was reported previously [26]. When disease onset was taken into account, weak biofilm production was associated with EOD exclusively for strains belonging to CCs 17 and 19, while neonates with EOD caused by strains belonging to lineages other than CC-17 and CC-19 were significantly more likely to produce strong biofilms. These data suggest different roles for biofilm formation in colonization and disease among the lineages. Because maternal transmission is frequently implicated in cases of EOD, strong biofilm production in the less virulent lineages may result in the transmission of greater bacterial densities. More studies, however, are needed to assess the role that biofilms play in both EOD and transmission. The association between weak biofilm production and invasive disease across all isolates is in accordance with results in *Streptococcus pneumoniae*, which demonstrated decreased pathogenicity in biofilm-associated cells explained by an altered transcriptome favoring colonization over invasiveness [51]. While our assay assessed optimal biofilm production in vitro, the plasticity of this trait due to environmental conditions encountered during colonization and pathogenesis was not explored. It remains possible that the biofilm production reported here may not reflect the ability of specific isolates to form biofilms in every environment. The elucidation of conditions that may trigger attachment and biofilm production is critical to determine the role of GBS biofilms in disease and colonization.

Decreased biofilm production within CC-17 and -19 strains is interesting given the protection conferred to biofilm-associated bacteria and the prior finding that these two lineages persisted better in women despite antibiotic treatment [11]. It is therefore possible that weak biofilm producers belonging to CCs 17 and 19 utilize different environmental cues to induce biofilm formation, or have distinct persistence strategies that do not rely on biofilms. In support of the former, a prior study found that exposure to acidic pH was an important factor for biofilm production by a subset of isolates belonging to ST-17 [25]. Because we observed no difference in T-HESC association levels between strong and weak biofilm producers overall, it is likely that biofilms are less important for host cell association. Biofilms take longer to form and cannot be reliably examined in these tissue culture assays, yet we expect that strong biofilm producers will have higher attachment levels and bacterial densities over time and hence, have a colonization advantage. We also expect these densities to vary across environments, genotypes, and individual isolates as was shown in our prior study between two CC-17 strains [52]. Indeed, it is possible that invasion of host cells is critical for strains that are not capable of forming strong biofilms, which may be more apparent in vivo. Although invasion frequencies were not calculated in this study, we expect them to be low given our prior findings [52] and importantly, differences would still be detectable using cell association frequencies that includes both the attached and invaded bacterial populations. These data are also in line with the epidemiological associations observed between CC-17 strains and invasive disease [14, 15] and our observation that invasive CC-17 strains had higher association levels compared to the colonizing CC-17 strains.

Together, these data highlight the phenotypic variability among GBS strains and support the hypothesis that strategies other than biofilm production are important for initial host cell attachment and persistence in some strains. Identifying alternative strategies requires further study, though variation in the ability to evade or survive within immune cells, invade the epithelia, and tolerate antibiotics, are all likely to be important. For those GBS strains that are capable of forming strong biofilms and also have enhanced association with host cells, it is possible that similar adherence mechanisms are used for each process. These mechanisms are likely to vary across genotypes and could be attributable to variation in different combinations of undefined or well-known surface proteins such as Lmb, Fbs, ScpB, Srr, and pili [18–24]. Indeed, strains from colonizing lineages have been shown to contain greater genetic diversity than invasive lineages like CC-17, which is well represented in our data, as variability in both biofilm production and host cell attachment was higher in colonizing lineages [53–55]. The observed differences in association levels between invasive and colonizing CC-17 strains, however, also suggest that variation between CC-17 strains exists. Additional studies are therefore needed to define the specific mechanisms of host cell attachment as well as biofilm production in diverse GBS strain populations; such studies will facilitate the identification of unique therapeutic or vaccine targets. Furthermore, because biofilms confer protection from antibiotics and immune system effectors, and contribute to the development of chronic infections in multiple bacterial pathogens, these findings posit biofilm production in GBS as clinically important for colonization in lineages other than CCs 17 and 19. Despite the generally weak biofilm production observed in disease-associated lineages, GBS colonization is an important risk factor for neonatal infections as well as opportunistic infections in susceptible individuals regardless of bacterial genotype. Hence, eradicating or thwarting biofilm production should be considered in the development of novel treatment and prevention strategies for GBS-mediated diseases.

## Conclusions

Quantifying biofilm production among 293 GBS strains from diverse sources demonstrated variation in the ability to form strong biofilms among strains belonging to different genotypes and from distinct sources. Those strains originating from bovines were capable of forming strong biofilms relative to strains from humans, though invasive versus colonizing human-derived strains belonging to CCs 17 and 19 were more likely to produce weak biofilms. Specific PI profiles and allelic variation within PI genes were also important for strong biofilm production, but no difference was observed in the ability of strong and weak biofilm producers to associate with T-HESCs. In all, these findings suggest that biofilm production is important for a subset of GBS strains and should be considered in the treatment of GBS-positive pregnant women to limit transmission to newborns.

## Methods

### Bacterial strains

A total of 293 GBS strains representing 73 STs and eight CCs were characterized in this study. A complete list of the strains evaluated can be found in Additional file 1: Table S1. Most strains were recovered from the blood or cerebral spinal fluid of neonates (invasive strains; *n* = 120) or vaginal/rectal swabs of pregnant women (colonizing strains; *n* = 88). Approval to characterize the de-identified bacterial isolates was provided by both the University of Calgary Ethics Board and Michigan State University Institutional Review Board. For comparison, 51 strains from quarter milk samples previously recovered from bovines with clinical or subclinical mastitis were characterized [56] and a reference set of 35 human-derived strains of varying STs and serotypes was included to compare biofilm production across phylogenetically distinct lineages. Reference strains included genome and control strains (*n* = 14) as well as ten strains each from adults with invasive disease and non-pregnant women. Except where otherwise indicated, GBS cultures were grown overnight in Todd-Hewitt (TH) broth at 37 **°**C with 5 % CO_2_. Strains were previously characterized for the PI type and allelic variation within the PI-2a backbone protein gene (*gbs59*) and the PI-2b adhesin gene (*san1519*) [41].

### Biofilm assays

Overnight cultures inoculated from freezer stocks were grown in TH broth, and then diluted 1:20 in fresh TH supplemented with 1 % glucose (THG). A total of 100 μl of the diluted culture was added to a 96-well plate with four technical replicates per strain. Cells were grown under static conditions at 37 °C with 5 % CO_2_ for 20 h. Following incubation, unattached bacteria were removed by washing twice with Phosphate Buffered Saline (PBS) (200ul), and attached bacteria were stained with 100 μl crystal violet for 10 min. Unbound crystal violet was removed by washing three times with PBS, and bound crystal violet was solubilized with 200 ul of 95 % ethanol. Biofilm production was quantified through absorbance readings (OD_595_) using a plate reader (Beckman Coulter, Inc.) and measurements were calculated as the sample value minus the media (blank) control. All assays were repeated at least three times with at least three technical replicates. All OD_595_ values above 1.8, the median value of all strains tested, represented strains that produce a strong biofilm. To determine this cutoff value, absorbance values were log-transformed and found to pass the D’Agostino and Pearson test of normality [57]. Chi-square (*χ*^2^) and the Fisher’s Exact test for sample sizes less than five were used to examine associations with biofilm production (weak versus strong) using SAS (version 9.3); a *P*-value < 0.05 was considered significant. Odds ratios (OR) and 95 % confidence intervals (95 % CI) were calculated to describe the univariate relationships. Multivariate analyses were conducted using logistic regression to identify predictors of strong biofilm production among the human-derived strains.

### Association with telomerase-immortalized human endometrial cells (T-HESC)

T-HESCs were decidualized as previously described [58] by growing the cells to ~50 % confluence and treating with 0.5 mM 8-bromo-cAMP (Sigma-Aldrich; St. Louis, MO) for three to six days. Decidualization was confirmed by examining the expression of prolactin and insulin-like growth factor (IGF) binding protein 1. Assays were not performed until the cells reached 100 % confluency; no part of the bottom of the well was exposed to avoid attachment of bacteria to the plastic plates. GBS strains were selected for testing based on phylogenetic lineage (CC) and biofilm phenotype with equal representation of weak and strong biofilm producers for each CC tested. An equal number of strains from cases of invasive disease and asymptomatic colonization were also evaluated when possible.

Bacterial strains were grown overnight in TH broth, washed once with PBS and resuspended in T-HESC infection medium, as previously described, except infecting inoculums were taken directly from overnight growth [52]. Host cells were washed three times with PBS prior to adding GBS at a multiplicity of infection (MOI) of one bacterial cell per host cell. After a 2 h incubation at 37 °C with 5 % CO_2_, 100 μL of supernatant was removed and serial diluted to determine final bacterial growth. Wells were then washed three times with PBS to remove non-adherent bacteria. To determine the number of associated bacteria, host cells were lysed with 0.1 % Triton X-100 (Sigma) for 30 min at 37 °C. Lysates were gently vortexed to further disrupt the host cells and liberate intracellular bacteria. After serial dilution, lysates were plated on THA, incubated overnight at 37 °C, and colony forming units (CFUs) were counted. All data were expressed as a percentage (number of associated divided by the total number of bacteria) after the two hour infection period. Individual assays were run in triplicate and each strain was tested at least three times.

## Additional file

Additional file 1: Strains examined in this study. (XLSX 27 kb)

## Abbreviations

cAMP: Cyclic Adenosine Monophosphate
CC: clonal complex
CFU: colony forming units
CI: confidence interval
DNA: deoxyribonucleic acid
EOD: early onset disease
FbsA: fibrinogen binding protein A
FbsB: fibrinogen binding protein B
GBS: Group B *Streptococcus*
IGF: insulin-like growth factor
Lac.2: lactose metabolism operon
Lmb: laminin-binding protein
LOD: late onset disease
MLST: multilocus sequence typing
OD: absorbance, or optical density
OR: odds ratio
PBS: Phosphate Buffered Saline
PI: Pilus Island
ScpB: Fibrinogen Binding C5a Peptidase
Srr-1: Serine Rich Repeat protein 1
Srr-2: Serine Rich Repeat protein 2
ST: sequence type
TH: Todd Hewitt Broth
T-HESC: Telomerase-Immortalized Human Endothelial Cells
THG: Todd Hewitt Broth plus 1 % glucose
